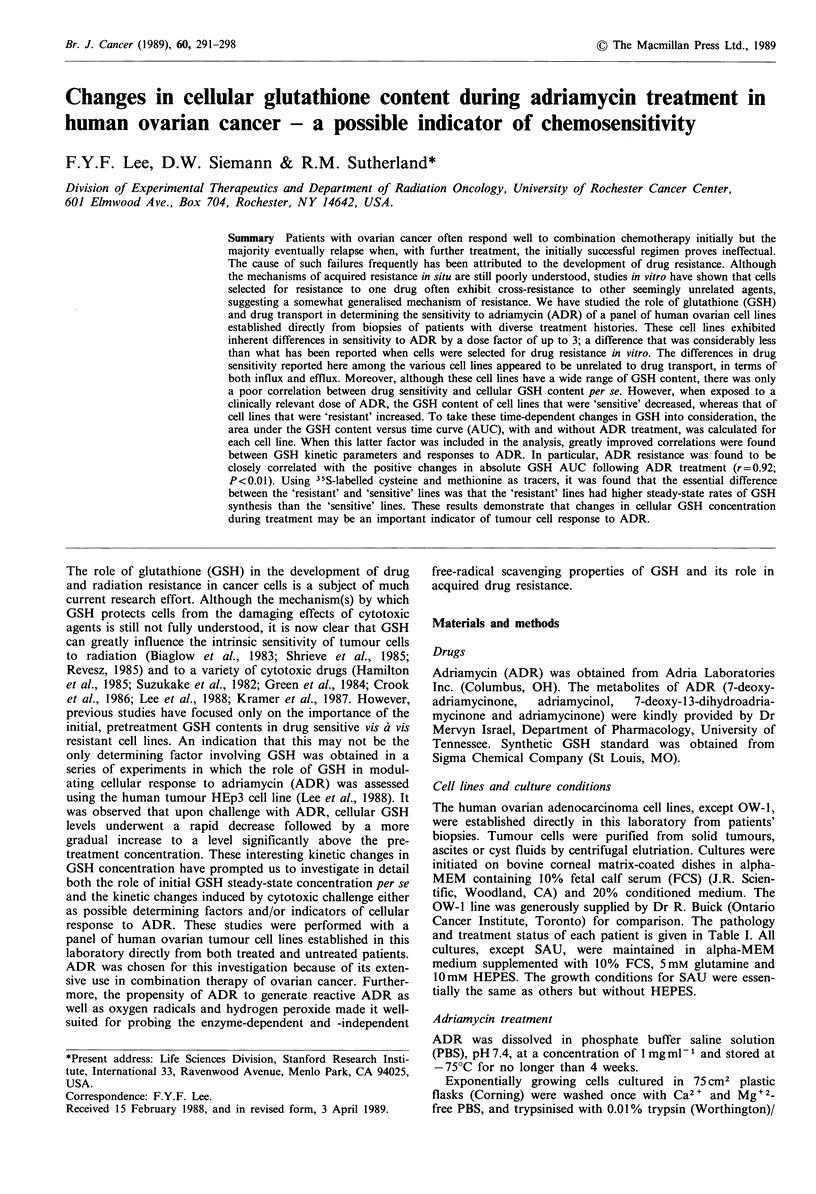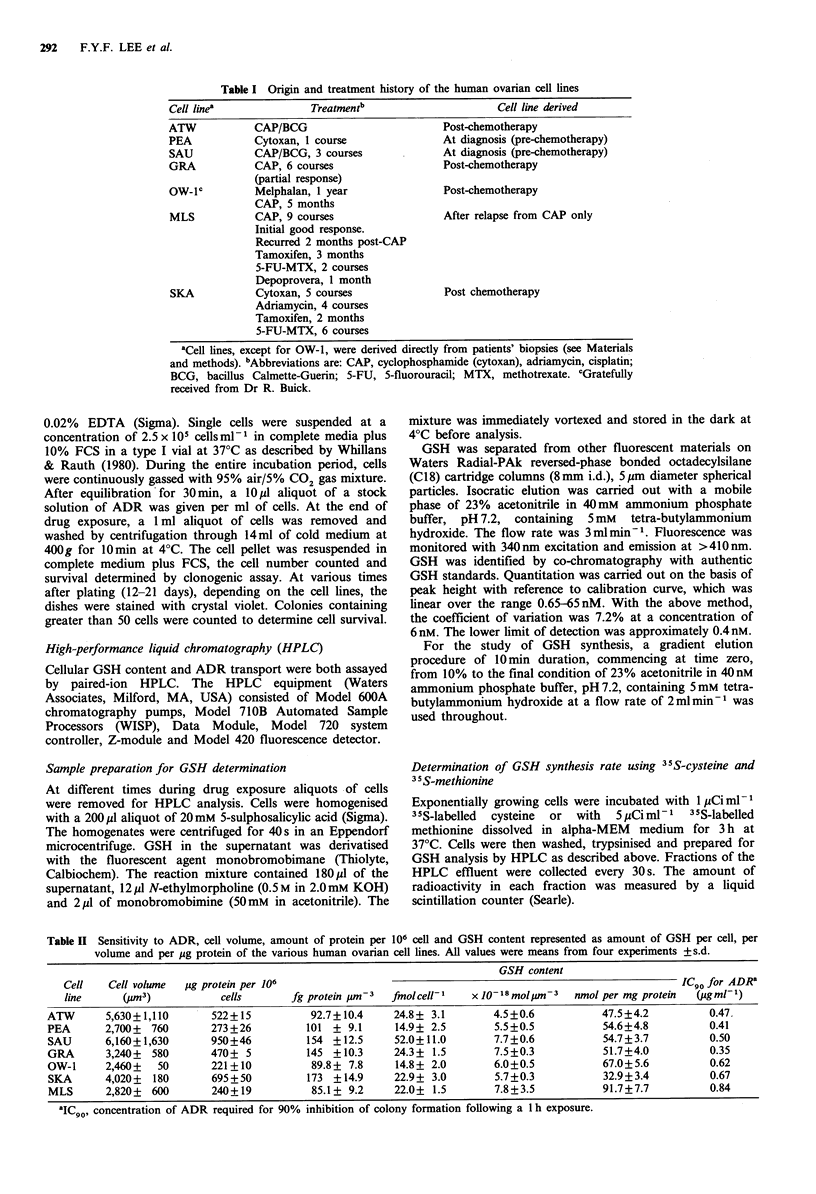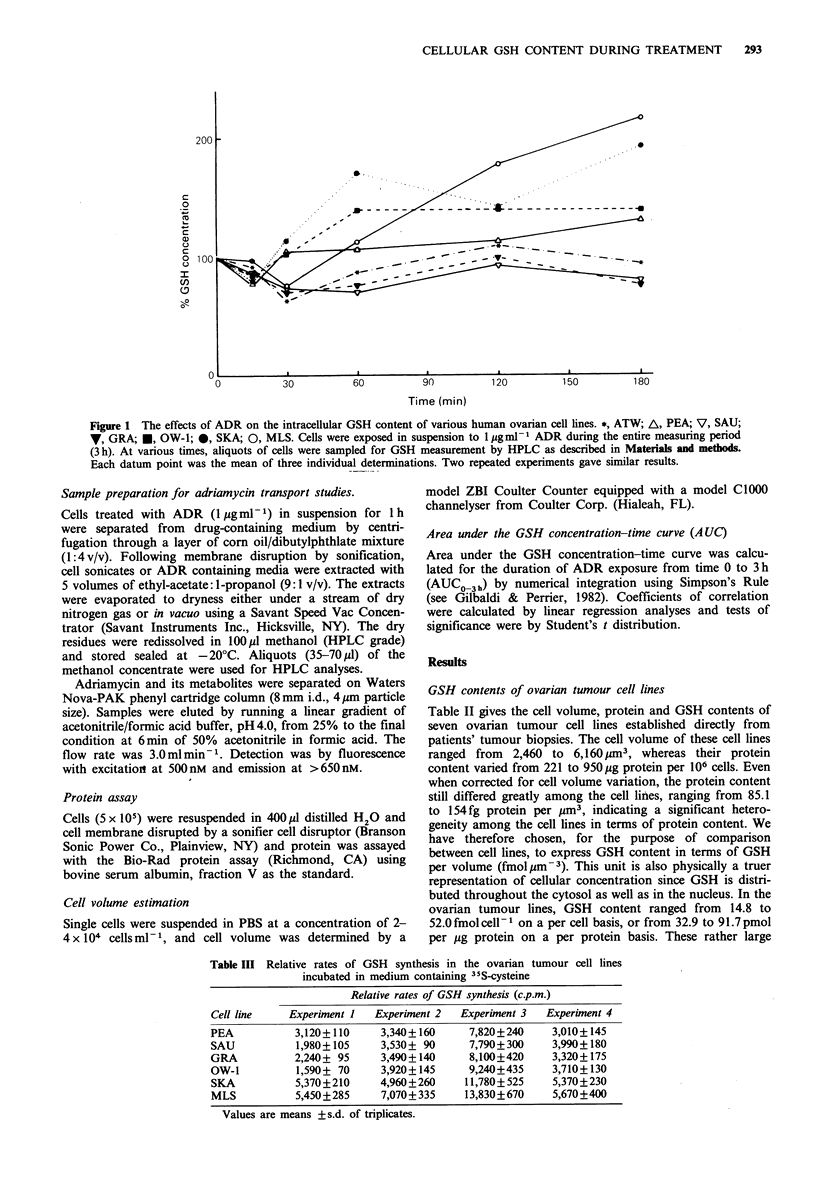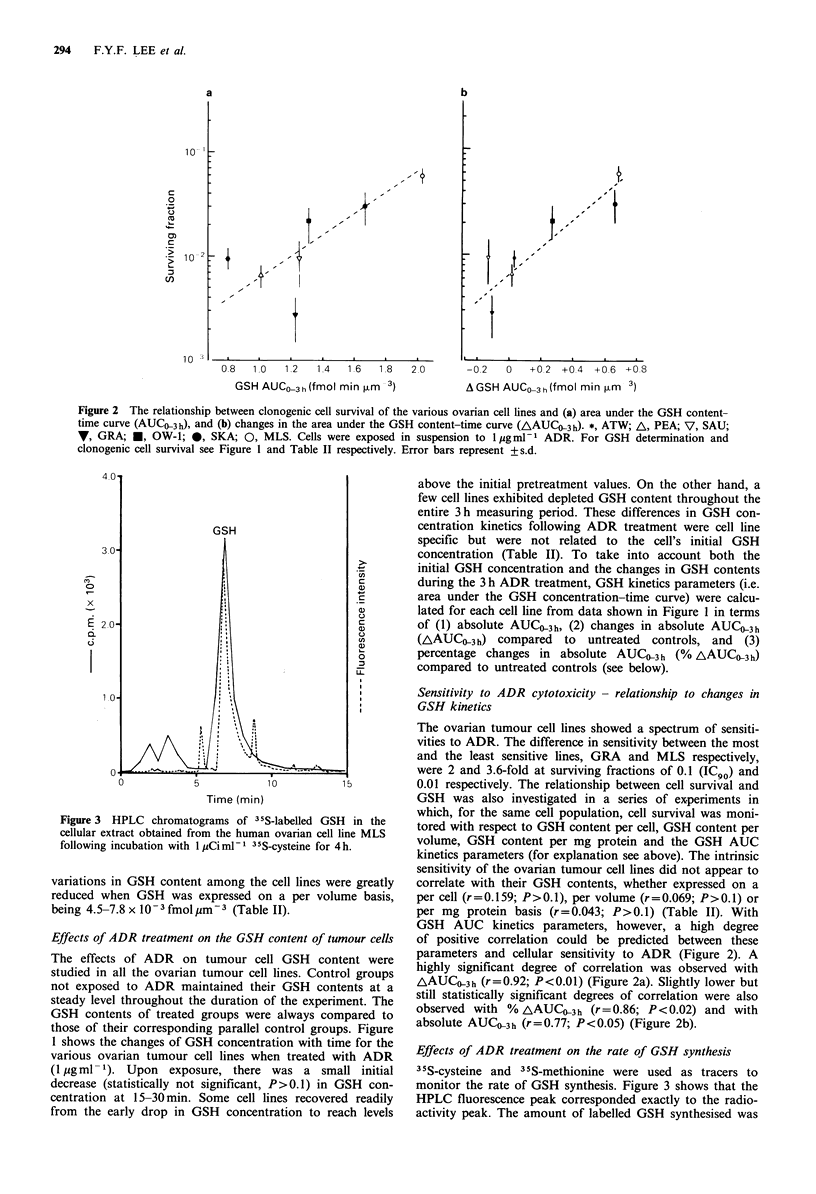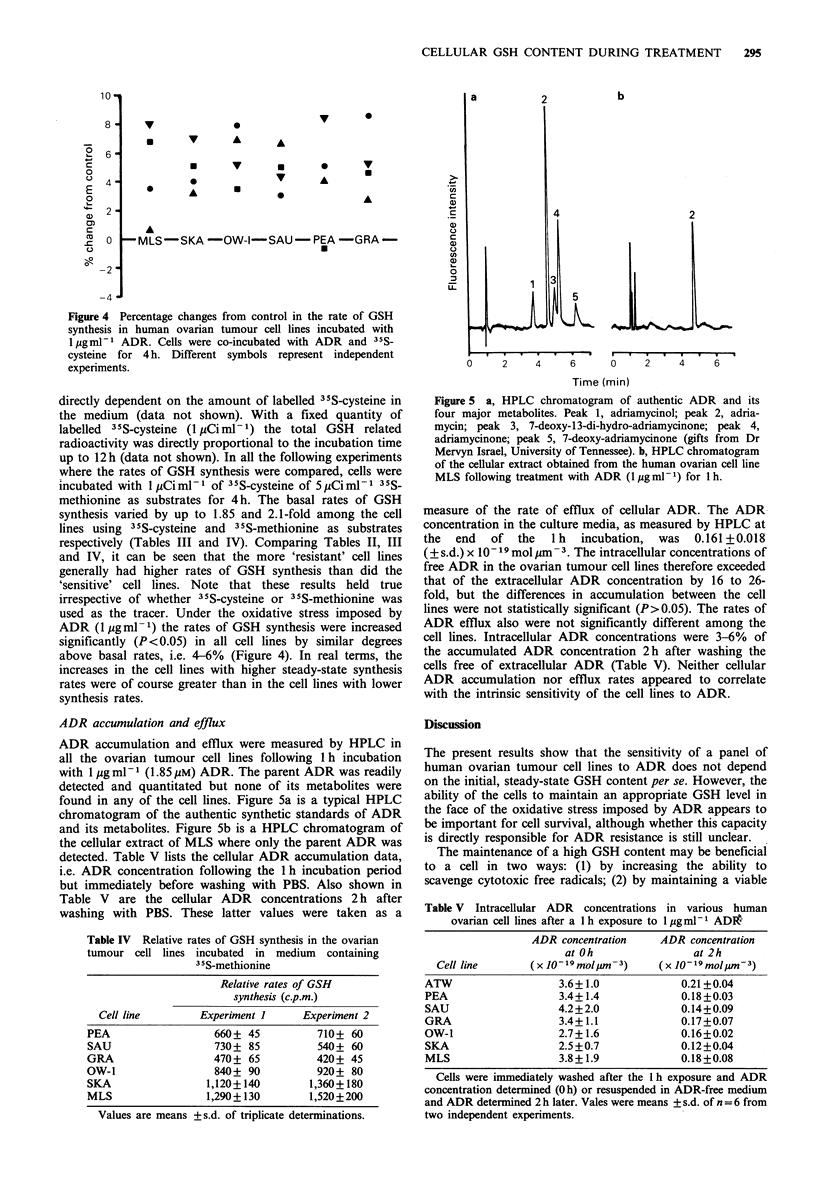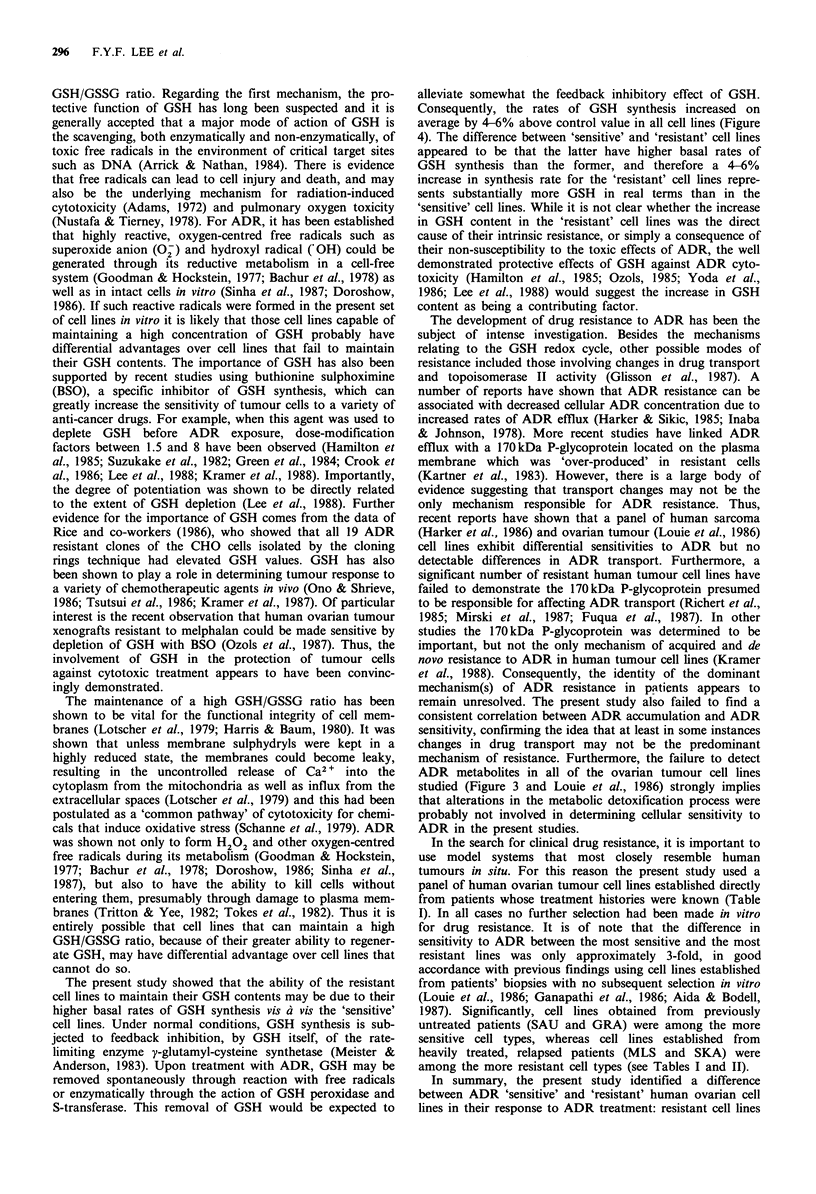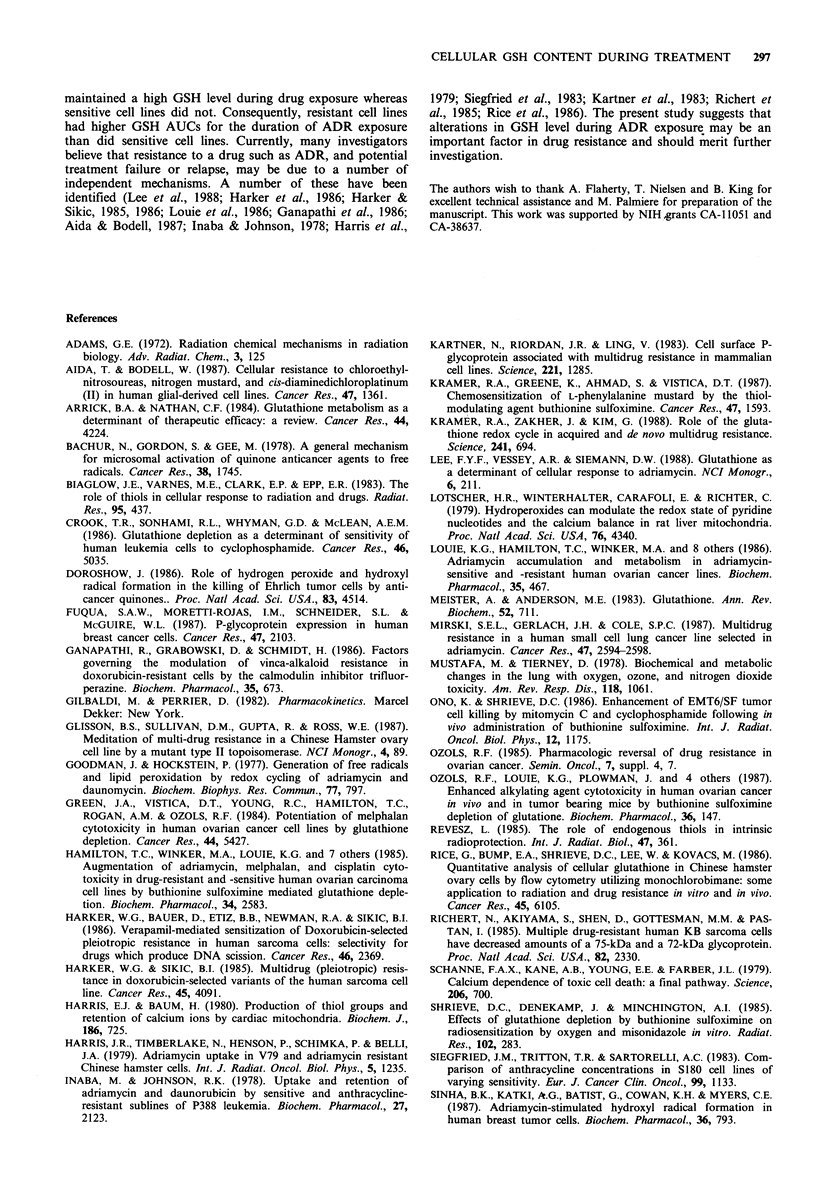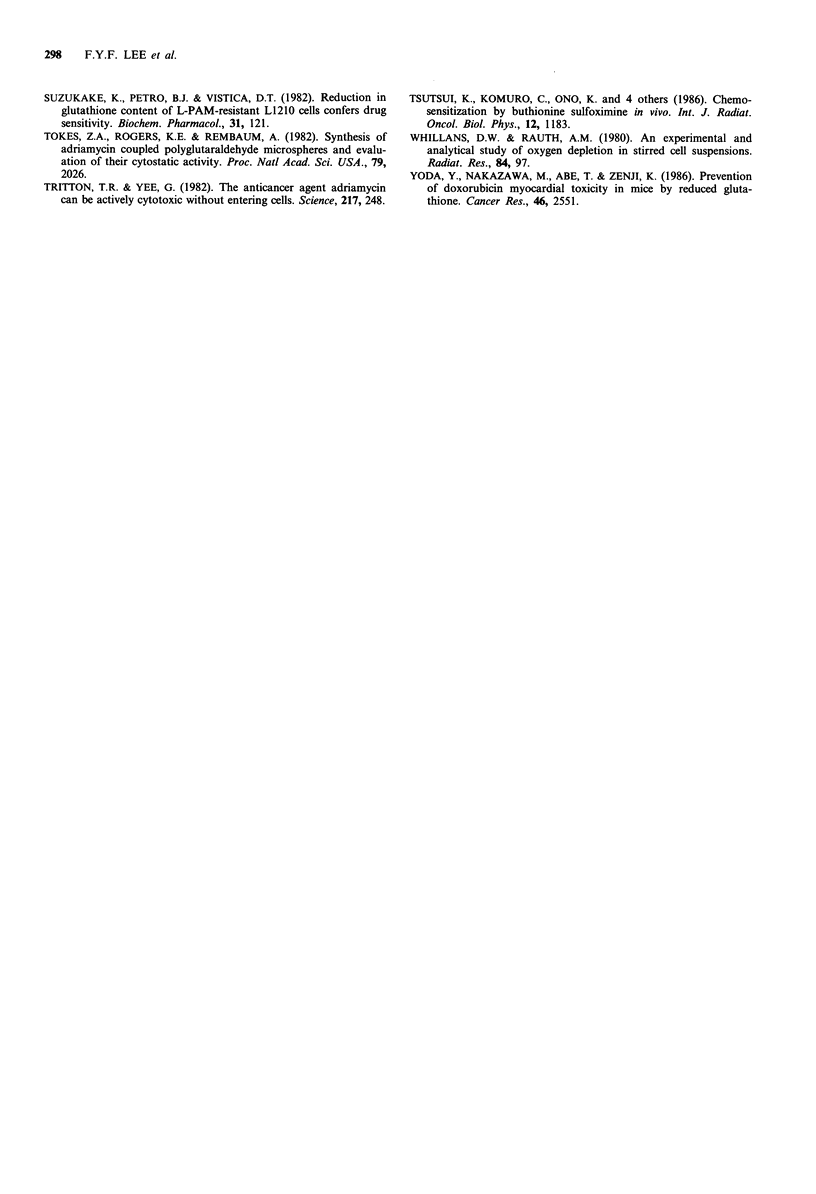# Changes in cellular glutathione content during adriamycin treatment in human ovarian cancer--a possible indicator of chemosensitivity.

**DOI:** 10.1038/bjc.1989.273

**Published:** 1989-09

**Authors:** F. Y. Lee, D. W. Siemann, R. M. Sutherland

**Affiliations:** Division of Experimental Therapeutics, University of Rochester Cancer Center, NY 14642.

## Abstract

Patients with ovarian cancer often respond well to combination chemotherapy initially but the majority eventually relapse when, with further treatment, the initially successful regimen proves ineffectual. The cause of such failures frequently has been attributed to the development of drug resistance. Although the mechanisms of acquired resistance in situ are still poorly understood, studies in vitro have shown that cells selected for resistance to one drug often exhibit cross-resistance to other seemingly unrelated agents, suggesting a somewhat generalised mechanism of resistance. We have studied the role of glutathione (GSH) and drug transport in determining the sensitivity to adriamycin (ADR) of a panel of human ovarian cell lines established directly from biopsies of patients with diverse treatment histories. These cell lines exhibited inherent differences in sensitivity to ADR by a dose factor of up to 3; a difference that was considerably less than what has been reported when cells were selected for drug resistance in vitro. The differences in drug sensitivity reported here among the various cell lines appeared to be unrelated to drug transport, in terms of both influx and efflux. Moreover, although these cell lines have a wide range of GSH content, there was only a poor correlation between drug sensitivity and cellular GSH content per se. However, when exposed to a clinically relevant dose of ADR, the GSH content of cell lines that were 'sensitive' decreased, whereas that of cell lines that were 'resistant' increased. To take these time-dependent changes in GSH into consideration, the area under the GSH content versus time curve (AUC), with and without ADR treatment, was calculated for each cell line. When this latter factor was included in the analysis, greatly improved correlations were found between GSH kinetic parameters and responses to ADR. In particular, ADR resistance was found to be closely correlated with the positive changes in absolute GSH AUC following ADR treatment (r = 0.92; P less than 0.01). Using 35S-labelled cysteine and methionine as tracers, it was found that the essential difference between the 'resistant' and 'sensitive' lines was that the 'resistant' lines had higher steady-state rates of GSH synthesis than the 'sensitive' lines. These results demonstrate that changes in cellular GSH concentration during treatment may be an important indicator of tumour cell response to ADR.


*Br. J. Cancer* (1989), **60**, 291–298 © The Macmillan Press Ltd., 1989

### Changes in cellular glutathione content during adriamycin treatment in human ovarian cancer – a possible indicator of chemosensitivity

F.Y.F. Lee, D.W. Siemann & R.M. Sutherland*

*Division of Experimental Therapeutics and Department of Radiation Oncology, University of Rochester Cancer Center, 601 Elmwood Ave., Box 704, Rochester, NY 14642, USA.*

**Summary** Patients with ovarian cancer often respond well to combination chemotherapy initially but the majority eventually relapse when, with further treatment, the initially successful regimen proves ineffectual. The cause of such failures frequently has been attributed to the development of drug resistance. Although the mechanisms of acquired resistance *in situ* are still poorly understood, studies *in vitro* have shown that cells selected for resistance to one drug often exhibit cross-resistance to other seemingly unrelated agents, suggesting a somewhat generalised mechanism of resistance. We have studied the role of glutathione (GSH) and drug transport in determining the sensitivity to adriamycin (ADR) of a panel of human ovarian cell lines established directly from biopsies of patients with diverse treatment histories. These cell lines exhibited inherent differences in sensitivity to ADR by a dose factor of up to 3; a difference that was considerably less than what has been reported when cells were selected for drug resistance *in vitro*. The differences in drug sensitivity reported here among the various cell lines appeared to be unrelated to drug transport, in terms of both influx and efflux. Moreover, although these cell lines have a wide range of GSH content, there was only a poor correlation between drug sensitivity and cellular GSH content *per se*. However, when exposed to a clinically relevant dose of ADR, the GSH content of cell lines that were 'sensitive' decreased, whereas that of cell lines that were 'resistant' increased. To take these time-dependent changes in GSH into consideration, the area under the GSH content versus time curve (AUC), with and without ADR treatment, was calculated for each cell line. When this latter factor was included in the analysis, greatly improved correlations were found between GSH kinetic parameters and responses to ADR. In particular, ADR resistance was found to be closely correlated with the positive changes in absolute GSH AUC following ADR treatment ($r = 0.92$; $P < 0.01$). Using $^{35}S$-labelled cysteine and methionine as tracers, it was found that the essential difference between the 'resistant' and 'sensitive' lines was that the 'resistant' lines had higher steady-state rates of GSH synthesis than the 'sensitive' lines. These results demonstrate that changes in cellular GSH concentration during treatment may be an important indicator of tumour cell response to ADR.

The role of glutathione (GSH) in the development of drug and radiation resistance in cancer cells is a subject of much current research effort. Although the mechanism(s) by which GSH protects cells from the damaging effects of cytotoxic agents is still not fully understood, it is now clear that GSH can greatly influence the intrinsic sensitivity of tumour cells to radiation (Biaglow *et al.*, 1983; Shrieve *et al.*, 1985; Revesz, 1985) and to a variety of cytotoxic drugs (Hamilton *et al.*, 1985; Suzukake *et al.*, 1982; Green *et al.*, 1984; Crook *et al.*, 1986; Lee *et al.*, 1988; Kramer *et al.*, 1987. However, previous studies have focused only on the importance of the initial, pretreatment GSH contents in drug sensitive *vis à vis* resistant cell lines. An indication that this may not be the only determining factor involving GSH was obtained in a series of experiments in which the role of GSH in modulating cellular response to adriamycin (ADR) was assessed using the human tumour HEp3 cell line (Lee *et al.*, 1988). It was observed that upon challenge with ADR, cellular GSH levels underwent a rapid decrease followed by a more gradual increase to a level significantly above the pretreatment concentration. These interesting kinetic changes in GSH concentration have prompted us to investigate in detail both the role of initial GSH steady-state concentration *per se* and the kinetic changes induced by cytotoxic challenge either as possible determining factors and/or indicators of cellular response to ADR. These studies were performed with a panel of human ovarian tumour cell lines established in this laboratory directly from both treated and untreated patients. ADR was chosen for this investigation because of its extensive use in combination therapy of ovarian cancer. Furthermore, the propensity of ADR to generate reactive ADR as well as oxygen radicals and hydrogen peroxide made it well-suited for probing the enzyme-dependent and -independent free-radical scavenging properties of GSH and its role in acquired drug resistance.

*Present address: Life Sciences Division, Stanford Research Institute, International 33, Ravenwood Avenue, Menlo Park, CA 94025, USA.
Correspondence: F.Y.F. Lee.
Received 15 February 1988, and in revised form, 3 April 1989.

### Materials and methods

### *Drugs*

Adriamycin (ADR) was obtained from Adria Laboratories Inc. (Columbus, OH). The metabolites of ADR (7-deoxy-adriamycinone, adriamycinol, 7-deoxy-13-dihydroadriamycinone and adriamycinone) were kindly provided by Dr Mervyn Israel, Department of Pharmacology, University of Tennessee. Synthetic GSH standard was obtained from Sigma Chemical Company (St Louis, MO).

### *Cell lines and culture conditions*

The human ovarian adenocarcinoma cell lines, except OW-1, were established directly in this laboratory from patients' biopsies. Tumour cells were purified from solid tumours, ascites or cyst fluids by centrifugal elutriation. Cultures were initiated on bovine corneal matrix-coated dishes in alpha-MEM containing 10% fetal calf serum (FCS) (J.R. Scientific, Woodland, CA) and 20% conditioned medium. The OW-1 line was generously supplied by Dr R. Buick (Ontario Cancer Institute, Toronto) for comparison. The pathology and treatment status of each patient is given in Table I. All cultures, except SAU, were maintained in alpha-MEM medium supplemented with 10% FCS, 5 mM glutamine and 10 mM HEPES. The growth conditions for SAU were essentially the same as others but without HEPES.

### *Adriamycin treatment*

ADR was dissolved in phosphate buffer saline solution (PBS), pH 7.4, at a concentration of $1\,mg\,ml^{-1}$ and stored at $-75°C$ for no longer than 4 weeks.

Exponentially growing cells cultured in $75\,cm^2$ plastic flasks (Corning) were washed once with $Ca^{2+}$ and $Mg^{+2}$-free PBS, and trypsinised with 0.01% trypsin (Worthington)/

**Table I** Origin and treatment history of the human ovarian cell lines

| *Cell line*[a] | *Treatment*[b] | *Cell line derived* |
|---|---|---|
| ATW | CAP/BCG | Post-chemotherapy |
| PEA | Cytoxan, 1 course | At diagnosis (pre-chemotherapy) |
| SAU | CAP/BCG, 3 courses | At diagnosis (pre-chemotherapy) |
| GRA | CAP, 6 courses (partial response) | Post-chemotherapy |
| OW-1[c] | Melphalan, 1 year CAP, 5 months | Post-chemotherapy |
| MLS | CAP, 9 courses Initial good response. Recurred 2 months post-CAP Tamoxifen, 3 months 5-FU-MTX, 2 courses Depoprovera, 1 month | After relapse from CAP only |
| SKA | Cytoxan, 5 courses Adriamycin, 4 courses Tamoxifen, 2 months 5-FU-MTX, 6 courses | Post chemotherapy |

[a]Cell lines, except for OW-1, were derived directly from patients' biopsies (see Materials and methods). [b]Abbreviations are: CAP, cyclophosphamide (cytoxan), adriamycin, cisplatin; BCG, bacillus Calmette-Guerin; 5-FU, 5-fluorouracil; MTX, methotrexate. [c]Gratefully received from Dr R. Buick.

0.02% EDTA (Sigma). Single cells were suspended at a concentration of $2.5 \times 10^5$ cells $ml^{-1}$ in complete media plus 10% FCS in a type I vial at 37°C as described by Whillans & Rauth (1980). During the entire incubation period, cells were continuously gassed with 95% air/5% $CO_2$ gas mixture. After equilibration for 30 min, a 10 $\mu$l aliquot of a stock solution of ADR was given per ml of cells. At the end of drug exposure, a 1 ml aliquot of cells was removed and washed by centrifugation through 14 ml of cold medium at 400 *g* for 10 min at 4°C. The cell pellet was resuspended in complete medium plus FCS, the cell number counted and survival determined by clonogenic assay. At various times after plating (12–21 days), depending on the cell lines, the dishes were stained with crystal violet. Colonies containing greater than 50 cells were counted to determine cell survival.

*High-performance liquid chromatography (HPLC)*

Cellular GSH content and ADR transport were both assayed by paired-ion HPLC. The HPLC equipment (Waters Associates, Milford, MA, USA) consisted of Model 600A chromatography pumps, Model 710B Automated Sample Processors (WISP), Data Module, Model 720 system controller, Z-module and Model 420 fluorescence detector.

*Sample preparation for GSH determination*

At different times during drug exposure aliquots of cells were removed for HPLC analysis. Cells were homogenised with a 200 $\mu$l aliquot of 20 mM 5-sulphosalicylic acid (Sigma). The homogenates were centrifuged for 40 s in an Eppendorf microcentrifuge. GSH in the supernatant was derivatised with the fluorescent agent monobromobimane (Thiolyte, Calbiochem). The reaction mixture contained 180 $\mu$l of the supernatant, 12 $\mu$l *N*-ethylmorpholine (0.5 M in 2.0 mM KOH) and 2 $\mu$l of monobromobimine (50 mM in acetonitrile). The mixture was immediately vortexed and stored in the dark at 4°C before analysis.

GSH was separated from other fluorescent materials on Waters Radial-PAk reversed-phase bonded octadecylsilane (C18) cartridge columns (8 mm i.d.), 5 $\mu$m diameter spherical particles. Isocratic elution was carried out with a mobile phase of 23% acetonitrile in 40 mM ammonium phosphate buffer, pH 7.2, containing 5 mM tetra-butylammonium hydroxide. The flow rate was 3 ml $min^{-1}$. Fluorescence was monitored with 340 nm excitation and emission at >410 nm. GSH was identified by co-chromatography with authentic GSH standards. Quantitation was carried out on the basis of peak height with reference to calibration curve, which was linear over the range 0.65–65 nM. With the above method, the coefficient of variation was 7.2% at a concentration of 6 nM. The lower limit of detection was approximately 0.4 nM.

For the study of GSH synthesis, a gradient elution procedure of 10 min duration, commencing at time zero, from 10% to the final condition of 23% acetonitrile in 40 nM ammonium phosphate buffer, pH 7.2, containing 5 mM tetrabutylammonium hydroxide at a flow rate of 2 ml $min^{-1}$ was used throughout.

*Determination of GSH synthesis rate using $^{35}S$-cysteine and $^{35}S$-methionine*

Exponentially growing cells were incubated with 1 $\mu$Ci $ml^{-1}$ $^{35}$S-labelled cysteine or with 5 $\mu$Ci $ml^{-1}$ $^{35}$S-labelled methionine dissolved in alpha-MEM medium for 3 h at 37°C. Cells were then washed, trypsinised and prepared for GSH analysis by HPLC as described above. Fractions of the HPLC effluent were collected every 30 s. The amount of radioactivity in each fraction was measured by a liquid scintillation counter (Searle).

**Table II** Sensitivity to ADR, cell volume, amount of protein per $10^6$ cell and GSH content represented as amount of GSH per cell, per volume and per $\mu$g protein of the various human ovarian cell lines. All values were means from four experiments ±s.d.

| *Cell line* | *Cell volume* ($\mu m^3$) | *$\mu$g protein per $10^6$ cells* | *fg protein $\mu m^{-3}$* | *GSH content* *fmol cell$^{-1}$* | *GSH content* $\times 10^{-18}$ *mol $\mu m^{-3}$* | *GSH content* *nmol per mg protein* | *$IC_{90}$ for ADR*[a] ($\mu g\, ml^{-1}$) |
|---|---|---|---|---|---|---|---|
| ATW | 5,630±1,110 | 522±15 | 92.7±10.4 | 24.8± 3.1 | 4.5±0.6 | 47.5±4.2 | 0.47 |
| PEA | 2,700± 760 | 273±26 | 101 ± 9.1 | 14.9± 2.5 | 5.5±0.5 | 54.6±4.8 | 0.41 |
| SAU | 6,160±1,630 | 950±46 | 154 ±12.5 | 52.0±11.0 | 7.7±0.6 | 54.7±3.7 | 0.50 |
| GRA | 3,240± 580 | 470± 5 | 145 ±10.3 | 24.3± 1.5 | 7.5±0.3 | 51.7±4.0 | 0.35 |
| OW-1 | 2,460± 50 | 221±10 | 89.8± 7.8 | 14.8± 2.0 | 6.0±0.5 | 67.0±5.6 | 0.62 |
| SKA | 4,020± 180 | 695±50 | 173 ±14.9 | 22.9± 3.0 | 5.7±0.3 | 32.9±3.4 | 0.67 |
| MLS | 2,820± 600 | 240±19 | 85.1± 9.2 | 22.0± 1.5 | 7.8±3.5 | 91.7±7.7 | 0.84 |

[a]$IC_{90}$, concentration of ADR required for 90% inhibition of colony formation following a 1 h exposure.

**Figure 1** The effects of ADR on the intracellular GSH content of various human ovarian cell lines. ∗, ATW; △, PEA; ▽, SAU; ▼, GRA; ■, OW-1; ●, SKA; ○, MLS. Cells were exposed in suspension to 1 $\mu g ml^{-1}$ ADR during the entire measuring period (3 h). At various times, aliquots of cells were sampled for GSH measurement by HPLC as described in **Materials and methods**. Each datum point was the mean of three individual determinations. Two repeated experiments gave similar results.

*Sample preparation for adriamycin transport studies.*

Cells treated with ADR (1 $\mu g ml^{-1}$) in suspension for 1 h were separated from drug-containing medium by centrifugation through a layer of corn oil/dibutylphthlate mixture (1:4 v/v). Following membrane disruption by sonification, cell sonicates or ADR containing media were extracted with 5 volumes of ethyl-acetate:1-propanol (9:1 v/v). The extracts were evaporated to dryness either under a stream of dry nitrogen gas or *in vacuo* using a Savant Speed Vac Concentrator (Savant Instruments Inc., Hicksville, NY). The dry residues were redissolved in 100 $\mu l$ methanol (HPLC grade) and stored sealed at −20°C. Aliquots (35–70 $\mu l$) of the methanol concentrate were used for HPLC analyses.

Adriamycin and its metabolites were separated on Waters Nova-PAK phenyl cartridge column (8 mm i.d., 4 $\mu m$ particle size). Samples were eluted by running a linear gradient of acetonitrile/formic acid buffer, pH 4.0, from 25% to the final condition at 6 min of 50% acetonitrile in formic acid. The flow rate was 3.0 ml $min^{-1}$. Detection was by fluorescence with excitation at 500 nM and emission at >650 nM.

*Protein assay*

Cells (5 × $10^5$) were resuspended in 400 $\mu l$ distilled $H_2O$ and cell membrane disrupted by a sonifier cell disruptor (Branson Sonic Power Co., Plainview, NY) and protein was assayed with the Bio-Rad protein assay (Richmond, CA) using bovine serum albumin, fraction V as the standard.

*Cell volume estimation*

Single cells were suspended in PBS at a concentration of 2–4 × $10^4$ cells $ml^{-1}$, and cell volume was determined by a model ZBI Coulter Counter equipped with a model C1000 channelyser from Coulter Corp. (Hialeah, FL).

*Area under the GSH concentration–time curve (AUC)*

Area under the GSH concentration–time curve was calculated for the duration of ADR exposure from time 0 to 3 h ($AUC_{0-3h}$) by numerical integration using Simpson's Rule (see Gilbaldi & Perrier, 1982). Coefficients of correlation were calculated by linear regression analyses and tests of significance were by Student's *t* distribution.

### Results

*GSH contents of ovarian tumour cell lines*

Table II gives the cell volume, protein and GSH contents of seven ovarian tumour cell lines established directly from patients' tumour biopsies. The cell volume of these cell lines ranged from 2,460 to 6,160 $\mu m^3$, whereas their protein content varied from 221 to 950 $\mu g$ protein per $10^6$ cells. Even when corrected for cell volume variation, the protein content still differed greatly among the cell lines, ranging from 85.1 to 154 fg protein per $\mu m^3$, indicating a significant heterogeneity among the cell lines in terms of protein content. We have therefore chosen, for the purpose of comparison between cell lines, to express GSH content in terms of GSH per volume (fmol $\mu m^{-3}$). This unit is also physically a truer representation of cellular concentration since GSH is distributed throughout the cytosol as well as in the nucleus. In the ovarian tumour lines, GSH content ranged from 14.8 to 52.0 fmol $cell^{-1}$ on a per cell basis, or from 32.9 to 91.7 pmol per $\mu g$ protein on a per protein basis. These rather large

**Table III** Relative rates of GSH synthesis in the ovarian tumour cell lines incubated in medium containing $^{35}S$-cysteine

| | *Relative rates of GSH synthesis (c.p.m.)* | | | |
|---|---|---|---|---|
| *Cell line* | *Experiment 1* | *Experiment 2* | *Experiment 3* | *Experiment 4* |
| PEA | 3,120±110 | 3,340±160 | 7,820±240 | 3,010±145 |
| SAU | 1,980±105 | 3,530± 90 | 7,790±300 | 3,990±180 |
| GRA | 2,240± 95 | 3,490±140 | 8,100±420 | 3,320±175 |
| OW-1 | 1,590± 70 | 3,920±145 | 9,240±435 | 3,710±130 |
| SKA | 5,370±210 | 4,960±260 | 11,780±525 | 5,370±230 |
| MLS | 5,450±285 | 7,070±335 | 13,830±670 | 5,670±400 |

Values are means ±s.d. of triplicates.

**Figure 2** The relationship between clonogenic cell survival of the various ovarian cell lines and (**a**) area under the GSH content–time curve ($AUC_{0-3h}$), and (**b**) changes in the area under the GSH content–time curve ($\Delta AUC_{0-3h}$). *, ATW; △, PEA; ▽, SAU; ▼, GRA; ■, OW-1; ●, SKA; ○, MLS. Cells were exposed in suspension to $1\,\mu g\,ml^{-1}$ ADR. For GSH determination and clonogenic cell survival see Figure 1 and Table II respectively. Error bars represent ±s.d.

**Figure 3** HPLC chromatograms of $^{35}S$-labelled GSH in the cellular extract obtained from the human ovarian cell line MLS following incubation with $1\,\mu Ci\,ml^{-1}$ $^{35}S$-cysteine for 4 h.

variations in GSH content among the cell lines were greatly reduced when GSH was expressed on a per volume basis, being $4.5–7.8 \times 10^{-3}\,fmol\,\mu m^{-3}$ (Table II).

*Effects of ADR treatment on the GSH content of tumour cells*

The effects of ADR on tumour cell GSH content were studied in all the ovarian tumour cell lines. Control groups not exposed to ADR maintained their GSH contents at a steady level throughout the duration of the experiment. The GSH contents of treated groups were always compared to those of their corresponding parallel control groups. Figure 1 shows the changes of GSH concentration with time for the various ovarian tumour cell lines when treated with ADR ($1\,\mu g\,ml^{-1}$). Upon exposure, there was a small initial decrease (statistically not significant, $P>0.1$) in GSH concentration at 15–30 min. Some cell lines recovered readily from the early drop in GSH concentration to reach levels above the initial pretreatment values. On the other hand, a few cell lines exhibited depleted GSH content throughout the entire 3 h measuring period. These differences in GSH concentration kinetics following ADR treatment were cell line specific but were not related to the cell's initial GSH concentration (Table II). To take into account both the initial GSH concentration and the changes in GSH contents during the 3 h ADR treatment, GSH kinetics parameters (i.e. area under the GSH concentration–time curve) were calculated for each cell line from data shown in Figure 1 in terms of (1) absolute $AUC_{0-3h}$, (2) changes in absolute $AUC_{0-3h}$ ($\Delta AUC_{0-3h}$) compared to untreated controls, and (3) percentage changes in absolute $AUC_{0-3h}$ (% $\Delta AUC_{0-3h}$) compared to untreated controls (see below).

*Sensitivity to ADR cytotoxicity – relationship to changes in GSH kinetics*

The ovarian tumour cell lines showed a spectrum of sensitivities to ADR. The difference in sensitivity between the most and the least sensitive lines, GRA and MLS respectively, were 2 and 3.6-fold at surviving fractions of 0.1 ($IC_{90}$) and 0.01 respectively. The relationship between cell survival and GSH was also investigated in a series of experiments in which, for the same cell population, cell survival was monitored with respect to GSH content per cell, GSH content per volume, GSH content per mg protein and the GSH AUC kinetics parameters (for explanation see above). The intrinsic sensitivity of the ovarian tumour cell lines did not appear to correlate with their GSH contents, whether expressed on a per cell ($r=0.159$; $P>0.1$), per volume ($r=0.069$; $P>0.1$) or per mg protein basis ($r=0.043$; $P>0.1$) (Table II). With GSH AUC kinetics parameters, however, a high degree of positive correlation could be predicted between these parameters and cellular sensitivity to ADR (Figure 2). A highly significant degree of correlation was observed with $\Delta AUC_{0-3h}$ ($r=0.92$; $P<0.01$) (Figure 2a). Slightly lower but still statistically significant degrees of correlation were also observed with % $\Delta AUC_{0-3h}$ ($r=0.86$; $P<0.02$) and with absolute $AUC_{0-3h}$ ($r=0.77$; $P<0.05$) (Figure 2b).

*Effects of ADR treatment on the rate of GSH synthesis*

$^{35}S$-cysteine and $^{35}S$-methionine were used as tracers to monitor the rate of GSH synthesis. Figure 3 shows that the HPLC fluorescence peak corresponded exactly to the radioactivity peak. The amount of labelled GSH synthesised was

**Figure 4** Percentage changes from control in the rate of GSH synthesis in human ovarian tumour cell lines incubated with 1 μg ml$^{-1}$ ADR. Cells were co-incubated with ADR and $^{35}$S-cysteine for 4 h. Different symbols represent independent experiments.

directly dependent on the amount of labelled $^{35}$S-cysteine in the medium (data not shown). With a fixed quantity of labelled $^{35}$S-cysteine (1 μCi ml$^{-1}$) the total GSH related radioactivity was directly proportional to the incubation time up to 12 h (data not shown). In all the following experiments where the rates of GSH synthesis were compared, cells were incubated with 1 μCi ml$^{-1}$ of $^{35}$S-cysteine of 5 μCi ml$^{-1}$ $^{35}$S-methionine as substrates for 4 h. The basal rates of GSH synthesis varied by up to 1.85 and 2.1-fold among the cell lines using $^{35}$S-cysteine and $^{35}$S-methionine as substrates respectively (Tables III and IV). Comparing Tables II, III and IV, it can be seen that the more 'resistant' cell lines generally had higher rates of GSH synthesis than did the 'sensitive' cell lines. Note that these results held true irrespective of whether $^{35}$S-cysteine or $^{35}$S-methionine was used as the tracer. Under the oxidative stress imposed by ADR (1 μg ml$^{-1}$) the rates of GSH synthesis were increased significantly ($P<0.05$) in all cell lines by similar degrees above basal rates, i.e. 4–6% (Figure 4). In real terms, the increases in the cell lines with higher steady-state synthesis rates were of course greater than in the cell lines with lower synthesis rates.

### *ADR accumulation and efflux*

ADR accumulation and efflux were measured by HPLC in all the ovarian tumour cell lines following 1 h incubation with 1 μg ml$^{-1}$ (1.85 μM) ADR. The parent ADR was readily detected and quantitated but none of its metabolites were found in any of the cell lines. Figure 5a is a typical HPLC chromatogram of the authentic synthetic standards of ADR and its metabolites. Figure 5b is a HPLC chromatogram of the cellular extract of MLS where only the parent ADR was detected. Table V lists the cellular ADR accumulation data, i.e. ADR concentration following the 1 h incubation period but immediately before washing with PBS. Also shown in Table V are the cellular ADR concentrations 2 h after washing with PBS. These latter values were taken as a

**Table IV** Relative rates of GSH synthesis in the ovarian tumour cell lines incubated in medium containing $^{35}$S-methionine

| Cell line | Relative rates of GSH synthesis (c.p.m.) Experiment 1 | Experiment 2 |
|---|---|---|
| PEA | 660 ± 45 | 710 ± 60 |
| SAU | 730 ± 85 | 540 ± 60 |
| GRA | 470 ± 65 | 420 ± 45 |
| OW-1 | 840 ± 90 | 920 ± 80 |
| SKA | 1,120 ± 140 | 1,360 ± 180 |
| MLS | 1,290 ± 130 | 1,520 ± 200 |

Values are means ± s.d. of triplicate determinations.

**Figure 5** **a**, HPLC chromatogram of authentic ADR and its four major metabolites. Peak 1, adriamycinol; peak 2, adriamycin; peak 3, 7-deoxy-13-di-hydro-adriamycinone; peak 4, adriamycinone; peak 5, 7-deoxy-adriamycinone (gifts from Dr Mervyn Israel, University of Tennessee). **b**, HPLC chromatogram of the cellular extract obtained from the human ovarian cell line MLS following treatment with ADR (1 μg ml$^{-1}$) for 1 h.

measure of the rate of efflux of cellular ADR. The ADR concentration in the culture media, as measured by HPLC at the end of the 1 h incubation, was 0.161 ± 0.018 (± s.d.) × 10$^{-19}$ mol μm$^{-3}$. The intracellular concentrations of free ADR in the ovarian tumour cell lines therefore exceeded that of the extracellular ADR concentration by 16 to 26-fold, but the differences in accumulation between the cell lines were not statistically significant ($P>0.05$). The rates of ADR efflux also were not significantly different among the cell lines. Intracellular ADR concentrations were 3–6% of the accumulated ADR concentration 2 h after washing the cells free of extracellular ADR (Table V). Neither cellular ADR accumulation nor efflux rates appeared to correlate with the intrinsic sensitivity of the cell lines to ADR.

### Discussion

The present results show that the sensitivity of a panel of human ovarian tumour cell lines to ADR does not depend on the initial, steady-state GSH content *per se*. However, the ability of the cells to maintain an appropriate GSH level in the face of the oxidative stress imposed by ADR appears to be important for cell survival, although whether this capacity is directly responsible for ADR resistance is still unclear.

The maintenance of a high GSH content may be beneficial to a cell in two ways: (1) by increasing the ability to scavenge cytotoxic free radicals; (2) by maintaining a viable

**Table V** Intracellular ADR concentrations in various human ovarian cell lines after a 1 h exposure to 1 μg ml$^{-1}$ ADR

| Cell line | ADR concentration at 0 h (× 10$^{-19}$ mol μm$^{-3}$) | ADR concentration at 2 h (× 10$^{-19}$ mol μm$^{-3}$) |
|---|---|---|
| ATW | 3.6 ± 1.0 | 0.21 ± 0.04 |
| PEA | 3.4 ± 1.4 | 0.18 ± 0.03 |
| SAU | 4.2 ± 2.0 | 0.14 ± 0.09 |
| GRA | 3.4 ± 1.1 | 0.17 ± 0.07 |
| OW-1 | 2.7 ± 1.6 | 0.16 ± 0.02 |
| SKA | 2.5 ± 0.7 | 0.12 ± 0.04 |
| MLS | 3.8 ± 1.9 | 0.18 ± 0.08 |

Cells were immediately washed after the 1 h exposure and ADR concentration determined (0 h) or resuspended in ADR-free medium and ADR determined 2 h later. Vales were means ± s.d. of $n=6$ from two independent experiments.

GSH/GSSG ratio. Regarding the first mechanism, the protective function of GSH has long been suspected and it is generally accepted that a major mode of action of GSH is the scavenging, both enzymatically and non-enzymatically, of toxic free radicals in the environment of critical target sites such as DNA (Arrick & Nathan, 1984). There is evidence that free radicals can lead to cell injury and death, and may also be the underlying mechanism for radiation-induced cytotoxicity (Adams, 1972) and pulmonary oxygen toxicity (Nustafa & Tierney, 1978). For ADR, it has been established that highly reactive, oxygen-centred free radicals such as superoxide anion ($O_2^-$) and hydroxyl radical ($^{\cdot}OH$) could be generated through its reductive metabolism in a cell-free system (Goodman & Hockstein, 1977; Bachur *et al.*, 1978) as well as in intact cells *in vitro* (Sinha *et al.*, 1987; Doroshow, 1986). If such reactive radicals were formed in the present set of cell lines *in vitro* it is likely that those cell lines capable of maintaining a high concentration of GSH probably have differential advantages over cell lines that fail to maintain their GSH contents. The importance of GSH has also been supported by recent studies using buthionine sulphoximine (BSO), a specific inhibitor of GSH synthesis, which can greatly increase the sensitivity of tumour cells to a variety of anti-cancer drugs. For example, when this agent was used to deplete GSH before ADR exposure, dose-modification factors between 1.5 and 8 have been observed (Hamilton *et al.*, 1985; Suzukake *et al.*, 1982; Green *et al.*, 1984; Crook *et al.*, 1986; Lee *et al.*, 1988; Kramer *et al.*, 1988). Importantly, the degree of potentiation was shown to be directly related to the extent of GSH depletion (Lee *et al.*, 1988). Further evidence for the importance of GSH comes from the data of Rice and co-workers (1986), who showed that all 19 ADR resistant clones of the CHO cells isolated by the cloning rings technique had elevated GSH values. GSH has also been shown to play a role in determining tumour response to a variety of chemotherapeutic agents *in vivo* (Ono & Shrieve, 1986; Tsutsui *et al.*, 1986; Kramer *et al.*, 1987). Of particular interest is the recent observation that human ovarian tumour xenografts resistant to melphalan could be made sensitive by depletion of GSH with BSO (Ozols *et al.*, 1987). Thus, the involvement of GSH in the protection of tumour cells against cytotoxic treatment appears to have been convincingly demonstrated.

The maintenance of a high GSH/GSSG ratio has been shown to be vital for the functional integrity of cell membranes (Lotscher *et al.*, 1979; Harris & Baum, 1980). It was shown that unless membrane sulphydryls were kept in a highly reduced state, the membranes could become leaky, resulting in the uncontrolled release of $Ca^{2+}$ into the cytoplasm from the mitochondria as well as influx from the extracellular spaces (Lotscher *et al.*, 1979) and this had been postulated as a 'common pathway' of cytotoxicity for chemicals that induce oxidative stress (Schanne *et al.*, 1979). ADR was shown not only to form $H_2O_2$ and other oxygen-centred free radicals during its metabolism (Goodman & Hockstein, 1977; Bachur *et al.*, 1978; Doroshow, 1986; Sinha *et al.*, 1987), but also to have the ability to kill cells without entering them, presumably through damage to plasma membranes (Tritton & Yee, 1982; Tokes *et al.*, 1982). Thus it is entirely possible that cell lines that can maintain a high GSH/GSSG ratio, because of their greater ability to regenerate GSH, may have differential advantage over cell lines that cannot do so.

The present study showed that the ability of the resistant cell lines to maintain their GSH contents may be due to their higher basal rates of GSH synthesis *vis à vis* the 'sensitive' cell lines. Under normal conditions, GSH synthesis is subjected to feedback inhibition, by GSH itself, of the rate-limiting enzyme γ-glutamyl-cysteine synthetase (Meister & Anderson, 1983). Upon treatment with ADR, GSH may be removed spontaneously through reaction with free radicals or enzymatically through the action of GSH peroxidase and S-transferase. This removal of GSH would be expected to alleviate somewhat the feedback inhibitory effect of GSH. Consequently, the rates of GSH synthesis increased on average by 4–6% above control value in all cell lines (Figure 4). The difference between 'sensitive' and 'resistant' cell lines appeared to be that the latter have higher basal rates of GSH synthesis than the former, and therefore a 4–6% increase in synthesis rate for the 'resistant' cell lines represents substantially more GSH in real terms than in the 'sensitive' cell lines. While it is not clear whether the increase in GSH content in the 'resistant' cell lines was the direct cause of their intrinsic resistance, or simply a consequence of their non-susceptibility to the toxic effects of ADR, the well demonstrated protective effects of GSH against ADR cytotoxicity (Hamilton *et al.*, 1985; Ozols, 1985; Yoda *et al.*, 1986; Lee *et al.*, 1988) would suggest the increase in GSH content as being a contributing factor.

The development of drug resistance to ADR has been the subject of intense investigation. Besides the mechanisms relating to the GSH redox cycle, other possible modes of resistance included those involving changes in drug transport and topoisomerase II activity (Glisson *et al.*, 1987). A number of reports have shown that ADR resistance can be associated with decreased cellular ADR concentration due to increased rates of ADR efflux (Harker & Sikic, 1985; Inaba & Johnson, 1978). More recent studies have linked ADR efflux with a 170 kDa P-glycoprotein located on the plasma membrane which was 'over-produced' in resistant cells (Kartner *et al.*, 1983). However, there is a large body of evidence suggesting that transport changes may not be the only mechanism responsible for ADR resistance. Thus, recent reports have shown that a panel of human sarcoma (Harker *et al.*, 1986) and ovarian tumour (Louie *et al.*, 1986) cell lines exhibit differential sensitivities to ADR but no detectable differences in ADR transport. Furthermore, a significant number of resistant human tumour cell lines have failed to demonstrate the 170 kDa P-glycoprotein presumed to be responsible for affecting ADR transport (Richert *et al.*, 1985; Mirski *et al.*, 1987; Fuqua *et al.*, 1987). In other studies the 170 kDa P-glycoprotein was determined to be important, but not the only mechanism of acquired and *de novo* resistance to ADR in human tumour cell lines (Kramer *et al.*, 1988). Consequently, the identity of the dominant mechanism(s) of ADR resistance in patients appears to remain unresolved. The present study also failed to find a consistent correlation between ADR accumulation and ADR sensitivity, confirming the idea that at least in some instances changes in drug transport may not be the predominant mechanism of resistance. Furthermore, the failure to detect ADR metabolites in all of the ovarian tumour cell lines studied (Figure 3 and Louie *et al.*, 1986) strongly implies that alterations in the metabolic detoxification process were probably not involved in determining cellular sensitivity to ADR in the present studies.

In the search for clinical drug resistance, it is important to use model systems that most closely resemble human tumours *in situ*. For this reason the present study used a panel of human ovarian tumour cell lines established directly from patients whose treatment histories were known (Table I). In all cases no further selection had been made *in vitro* for drug resistance. It is of note that the difference in sensitivity to ADR between the most sensitive and the most resistant lines was only approximately 3-fold, in good accordance with previous findings using cell lines established from patients' biopsies with no subsequent selection *in vitro* (Louie *et al.*, 1986; Ganapathi *et al.*, 1986; Aida & Bodell, 1987). Significantly, cell lines obtained from previously untreated patients (SAU and GRA) were among the more sensitive cell types, whereas cell lines established from heavily treated, relapsed patients (MLS and SKA) were among the more resistant cell types (see Tables I and II).

In summary, the present study identified a difference between ADR 'sensitive' and 'resistant' human ovarian cell lines in their response to ADR treatment: resistant cell lines maintained a high GSH level during drug exposure whereas sensitive cell lines did not. Consequently, resistant cell lines had higher GSH AUCs for the duration of ADR exposure than did sensitive cell lines. Currently, many investigators believe that resistance to a drug such as ADR, and potential treatment failure or relapse, may be due to a number of independent mechanisms. A number of these have been identified (Lee *et al.*, 1988; Harker *et al.*, 1986; Harker & Sikic, 1985, 1986; Louie *et al.*, 1986; Ganapathi *et al.*, 1986; Aida & Bodell, 1987; Inaba & Johnson, 1978; Harris *et al.*, 1979; Siegfried *et al.*, 1983; Kartner *et al.*, 1983; Richert *et al.*, 1985; Rice *et al.*, 1986). The present study suggests that alterations in GSH level during ADR exposure may be an important factor in drug resistance and should merit further investigation.

The authors wish to thank A. Flaherty, T. Nielsen and B. King for excellent technical assistance and M. Palmiere for preparation of the manuscript. This work was supported by NIH grants CA-11051 and CA-38637.

### References

ADAMS, G.E. (1972). Radiation chemical mechanisms in radiation biology. *Adv. Radiat. Chem.*, **3**, 125

AIDA, T. & BODELL, W. (1987). Cellular resistance to chloroethylnitrosoureas, nitrogen mustard, and *cis*-diaminedichloroplatinum (II) in human glial-derived cell lines. *Cancer Res.*, **47**, 1361.

ARRICK, B.A. & NATHAN, C.F. (1984). Glutathione metabolism as a determinant of therapeutic efficacy: a review. *Cancer Res.*, **44**, 4224.

BACHUR, N., GORDON, S. & GEE, M. (1978). A general mechanism for microsomal activation of quinone anticancer agents to free radicals. *Cancer Res.*, **38**, 1745.

BIAGLOW, J.E., VARNES, M.E., CLARK, E.P. & EPP, E.R. (1983). The role of thiols in cellular response to radiation and drugs. *Radiat. Res.*, **95**, 437.

CROOK, T.R., SONHAMI, R.L., WHYMAN, G.D. & McLEAN, A.E.M. (1986). Glutathione depletion as a determinant of sensitivity of human leukemia cells to cyclophosphamide. *Cancer Res.*, **46**, 5035.

DOROSHOW, J. (1986). Role of hydrogen peroxide and hydroxyl radical formation in the killing of Ehrlich tumor cells by anticancer quinones.. *Proc. Natl Acad. Sci. USA.*, **83**, 4514.

FUQUA, S.A.W., MORETTI-ROJAS, I.M., SCHNEIDER, S.L. & McGUIRE, W.L. (1987). P-glycoprotein expression in human breast cancer cells. *Cancer Res.*, **47**, 2103.

GANAPATHI, R., GRABOWSKI, D. & SCHMIDT, H. (1986). Factors governing the modulation of vinca-alkaloid resistance in doxorubicin-resistant cells by the calmodulin inhibitor trifluorperazine. *Biochem. Pharmacol.*, **35**, 673.

GILBALDI, M. & PERRIER, D. (1982). *Pharmacokinetics*. Marcel Dekker: New York.

GLISSON, B.S., SULLIVAN, D.M., GUPTA, R. & ROSS, W.E. (1987). Meditation of multi-drug resistance in a Chinese Hamster ovary cell line by a mutant type II topoisomerase. *NCI Monogr.*, **4**, 89.

GOODMAN, J. & HOCKSTEIN, P. (1977). Generation of free radicals and lipid peroxidation by redox cycling of adriamycin and daunomycin. *Biochem. Biophys. Res. Commun.*, **77**, 797.

GREEN, J.A., VISTICA, D.T., YOUNG, R.C., HAMILTON, T.C., ROGAN, A.M. & OZOLS, R.F. (1984). Potentiation of melphalan cytotoxicity in human ovarian cancer cell lines by glutathione depletion. *Cancer Res.*, **44**, 5427.

HAMILTON, T.C., WINKER, M.A., LOUIE, K.G. and 7 others (1985). Augmentation of adriamycin, melphalan, and cisplatin cytotoxicity in drug-resistant and -sensitive human ovarian carcinoma cell lines by buthionine sulfoximine mediated glutathione depletion. *Biochem. Pharmacol.*, **34**, 2583.

HARKER, W.G., BAUER, D., ETIZ, B.B., NEWMAN, R.A. & SIKIC, B.I. (1986). Verapamil-mediated sensitization of Doxorubicin-selected pleiotropic resistance in human sarcoma cells: selectivity for drugs which produce DNA scission. *Cancer Res.*, **46**, 2369.

HARKER, W.G. & SIKIC, B.I. (1985). Multidrug (pleiotropic) resistance in doxorubicin-selected variants of the human sarcoma cell line. *Cancer Res.*, **45**, 4091.

HARRIS, E.J. & BAUM, H. (1980). Production of thiol groups and retention of calcium ions by cardiac mitochondria. *Biochem. J.*, **186**, 725.

HARRIS, J.R., TIMBERLAKE, N., HENSON, P., SCHIMKA, P. & BELLI, J.A. (1979). Adriamycin uptake in V79 and adriamycin resistant Chinese hamster cells. *Int. J. Radiat. Oncol. Biol. Phys.*, **5**, 1235.

INABA, M. & JOHNSON, R.K. (1978). Uptake and retention of adriamycin and daunorubicin by sensitive and anthracycline-resistant sublines of P388 leukemia. *Biochem. Pharmacol.*, **27**, 2123.

KARTNER, N., RIORDAN, J.R. & LING, V. (1983). Cell surface P-glycoprotein associated with multidrug resistance in mammalian cell lines. *Science*, **221**, 1285.

KRAMER, R.A., GREENE, K., AHMAD, S. & VISTICA, D.T. (1987). Chemosensitization of L-phenylalanine mustard by the thiol-modulating agent buthionine sulfoximine. *Cancer Res.*, **47**, 1593.

KRAMER, R.A., ZAKHER, J. & KIM, G. (1988). Role of the glutathione redox cycle in acquired and *de novo* multidrug resistance. *Science*, **241**, 694.

LEE, F.Y.F., VESSEY, A.R. & SIEMANN, D.W. (1988). Glutathione as a determinant of cellular response to adriamycin. *NCI Monogr.*, **6**, 211.

LOTSCHER, H.R., WINTERHALTER, CARAFOLI, E. & RICHTER, C. (1979). Hydroperoxides can modulate the redox state of pyridine nucleotides and the calcium balance in rat liver mitochondria. *Proc. Natl Acad. Sci. USA*, **76**, 4340.

LOUIE, K.G., HAMILTON, T.C., WINKER, M.A. and 8 others (1986). Adriamycin accumulation and metabolism in adriamycin-sensitive and -resistant human ovarian cancer lines. *Biochem. Pharmacol.*, **35**, 467.

MEISTER, A. & ANDERSON, M.E. (1983). Glutathione. *Ann. Rev. Biochem.*, **52**, 711.

MIRSKI, S.E.L., GERLACH, J.H. & COLE, S.P.C. (1987). Multidrug resistance in a human small cell lung cancer line selected in adriamycin. *Cancer Res.*, **47**, 2594–2598.

MUSTAFA, M. & TIERNEY, D. (1978). Biochemical and metabolic changes in the lung with oxygen, ozone, and nitrogen dioxide toxicity. *Am. Rev. Resp. Dis.*, **118**, 1061.

ONO, K. & SHRIEVE, D.C. (1986). Enhancement of EMT6/SF tumor cell killing by mitomycin C and cyclophosphamide following *in vivo* administration of buthionine sulfoximine. *Int. J. Radiat. Oncol. Biol. Phys.*, **12**, 1175.

OZOLS, R.F. (1985). Pharmacologic reversal of drug resistance in ovarian cancer. *Semin. Oncol.*, **7**, suppl. 4, 7.

OZOLS, R.F., LOUIE, K.G., PLOWMAN, J. and 4 others (1987). Enhanced alkylating agent cytotoxicity in human ovarian cancer *in vivo* and in tumor bearing mice by buthionine sulfoximine depletion of glutatione. *Biochem. Pharmacol.*, **36**, 147.

REVESZ, L. (1985). The role of endogenous thiols in intrinsic radioprotection. *Int. J. Radiat. Biol.*, **47**, 361.

RICE, G., BUMP, E.A., SHRIEVE, D.C., LEE, W. & KOVACS, M. (1986). Quantitative analysis of cellular glutathione in Chinese hamster ovary cells by flow cytometry utilizing monochlorobimane: some application to radiation and drug resistance *in vitro* and *in vivo*. *Cancer Res.*, **45**, 6105.

RICHERT, N., AKIYAMA, S., SHEN, D., GOTTESMAN, M.M. & PASTAN, I. (1985). Multiple drug-resistant human KB sarcoma cells have decreased amounts of a 75-kDa and a 72-kDa glycoprotein. *Proc. Natl Acad. Sci. USA.*, **82**, 2330.

SCHANNE, F.A.X., KANE, A.B., YOUNG, E.E. & FARBER, J.L. (1979). Calcium dependence of toxic cell death: a final pathway. *Science*, **206**, 700.

SHRIEVE, D.C., DENEKAMP, J. & MINCHINGTON, A.I. (1985). Effects of glutathione depletion by buthionine sulfoximine on radiosensitization by oxygen and misonidazole *in vitro*. *Radiat. Res.*, **102**, 283.

SIEGFRIED, J.M., TRITTON, T.R. & SARTORELLI, A.C. (1983). Comparison of anthracycline concentrations in S180 cell lines of varying sensitivity. *Eur. J. Cancer Clin. Oncol.*, **99**, 1133.

SINHA, B.K., KATKI, A.G., BATIST, G., COWAN, K.H. & MYERS, C.E. (1987). Adriamycin-stimulated hydroxyl radical formation in human breast tumor cells. *Biochem. Pharmacol.*, **36**, 793.

SUZUKAKE, K., PETRO, B.J. & VISTICA, D.T. (1982). Reduction in glutathione content of L-PAM-resistant L1210 cells confers drug sensitivity. *Biochem. Pharmacol.*, **31**, 121.

TOKES, Z.A., ROGERS, K.E. & REMBAUM, A. (1982). Synthesis of adriamycin coupled polyglutaraldehyde microspheres and evaluation of their cytostatic activity. *Proc. Natl Acad. Sci. USA.*, **79**, 2026.

TRITTON, T.R. & YEE, G. (1982). The anticancer agent adriamycin can be actively cytotoxic without entering cells. *Science*, **217**, 248.

TSUTSUI, K., KOMURO, C., ONO, K. and 4 others (1986). Chemosensitization by buthionine sulfoximine *in vivo*. *Int. J. Radiat. Oncol. Biol. Phys.*, **12**, 1183.

WHILLANS, D.W. & RAUTH, A.M. (1980). An experimental and analytical study of oxygen depletion in stirred cell suspensions. *Radiat. Res.*, **84**, 97.

YODA, Y., NAKAZAWA, M., ABE, T. & ZENJI, K. (1986). Prevention of doxorubicin myocardial toxicity in mice by reduced glutathione. *Cancer Res.*, **46**, 2551.